# A Cationic Tetraphenylethene as a Light-Up Supramolecular Probe for DNA G-Quadruplexes

**DOI:** 10.3389/fchem.2019.00493

**Published:** 2019-07-11

**Authors:** Clément Kotras, Mathieu Fossépré, Maxime Roger, Virginie Gervais, Sébastien Richeter, Philippe Gerbier, Sébastien Ulrich, Mathieu Surin, Sébastien Clément

**Affiliations:** ^1^ICGM Institut Charles Gerhardt Montpellier, UMR 5253, CNRS, Université de Montpellier, ENSCM, Montpellier, France; ^2^Laboratory for Chemistry of Novel Materials, Center of Innovation and Research in Materials and Polymers, University of Mons-UMONS, Mons, Belgium; ^3^Institut de Pharmacologie et de Biologie Structurale, CNRS, IPBS, Université de Toulouse, Toulouse, France; ^4^Institut des Biomolécules Max Mousseron, IBMM, UMR 5247, CNRS, Université de Montpellier, ENSCM, Montpellier, France

**Keywords:** tetraphenylethene, G-quadruplexes, fluorescence, light-up probe, supramolecular

## Abstract

Guanine-quadruplexes (G4s) are targets for anticancer therapeutics. In this context, human telomeric DNA (HT-DNA) that can fold into G4s sequences are of particular interest, and their stabilization with small molecules through a visualizable process has become a challenge. As a new type of ligand for HT-G4, we designed a tetraimidazolium tetraphenylethene (**TPE-Im**) as a water-soluble light-up G4 probe. We study its G4-binding properties with HT-DNA by UV-Visible absorption, circular dichroism and fluorescence spectroscopies, which provide insights into the interactions between **TPE-Im** and G4-DNA. Remarkably, **TPE-Im** shows a strong fluorescence enhancement and large shifts upon binding to G4, which is valuable for detecting G4s. The association constants for the **TPE-Im**/G4 complex were evaluated in different solution conditions via isothermal titration calorimetry (ITC), and its binding modes were explored by molecular modeling showing a groove-binding mechanism. The stabilization of G4 by **TPE-Im** has been assessed by Fluorescence Resonance Energy Transfer (FRET) melting assays, which show a strong stabilization (Δ*T*_1/2_ around +20°C), together with a specificity toward G4 with respect to double-stranded DNA.

## Introduction

G-quadruplexes (G4s) are secondary structures of DNA, formed by specific guanine-rich sequences in presence of monovalent cations. Guanine-rich sequences can self-assemble into square planar networks (called G-quartets) via Hoogsteen hydrogen-bonds, and these G-quartets can stack on top of each other, stabilized by monovalent alkali cations, to form G-quadruplexes (G4s) (Davis and Spada, 2007; Bochman et al., 2012; Doluca et al., 2013). Notably, it has been demonstrated that G4s are formed in human telomeres, made of DNA sequence d(TTAGGG)n (Balasubramanian and Neidle, 2009; Phan, 2010; Wu and Brosh, 2010; Lam et al., 2013; Hänsel-Hertsch et al., 2017). Telomeres are non-coding regions at the end of chromosomes that protect chromosomal ends from fusion during replication of DNA. A specific enzyme, the telomerase, add repetitions of d(TTAGGG)n at the end of telomeres, and it has been shown that in most cancer cells, this enzyme is overexpressed. The normal expression of telomerase into cells allows protection of coding genes during the cell life, whereas when telomerase is overexpressed, the cell can no longer enter into senescence and start multiplying in a non-controlled manner, causing tumor growth (Phan, 2010; Wu and Brosh, 2010; Biffi et al., 2013; Maji and Bhattacharya, 2014). The challenge that emerge from these hypotheses in anti-cancer drug design is designing molecules that can bind/stabilize G4s and inhibit the telomerase activity in tumor cells (Monchaud and Teulade-Fichou, 2008; Balasubramanian and Neidle, 2009; Collie and Parkinson, 2011; Tucker et al., 2012; Neidle, 2017; Asamitsu et al., 2019). A large number of small molecules, such as porphyrin, perylene, and naphthalene diimide derivatives, have been prepared and demonstrated their ability to bind G4 (Zhao et al., 2013; Golub et al., 2015; Rubio-Magnieto et al., 2015). We have notably studied the interaction between imidazolium- or pyridinium-based tetracationic porphyrins and demonstrated their strong binding with G4s, and their selectivity to G4 over double-stranded DNA (dsDNA) (Rubio-Magnieto et al., 2015). Indeed, porphyrins have interesting UV-Vis absorption properties, but their fluorescence intensities in interaction with G4 are weak, hampering their use as fluorescent probes.

**Scheme 1:**
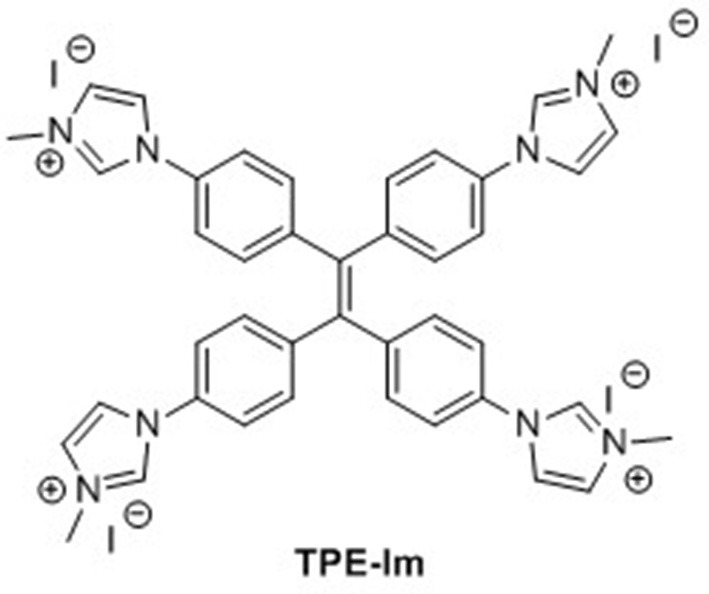
Chemical structure of **TPE-Im**.

With the aim of evolving toward G4-DNA fluorescent probes, we synthesized a cationic tetraphenylethene (TPE) derivative, as recently reported by Hahn and coworkers (Sinha et al., 2017). The TPE core was selected due to its appropriate size in comparison to intramolecular human telomeric G4s (Hong et al., 2010). To promote multi-site interactions and solubility in aqueous media, four cationic groups were introduced into the TPE core as it has been achieved with porphyrins by us and others (Flynn et al., 1999; Trommel and Marzilli, 2001; Rubio-Magnieto et al., 2015). A remarkable characteristic of TPEs is their peculiar fluorescence properties: TPEs are poorly fluorescent when molecularly dissolved in solution due to the possible intramolecular rotations which lead to deexcitation through non-radiative pathways. However, when TPEs aggregate, a large increase of the fluorescence is noted because of the restriction of its intramolecular rotations by the aggregate formation (Mei et al., 2014; Yang et al., 2016). These Aggregation-Induced Emission (AIE) properties of TPE motivated us to conceive a “light-up” probe to stabilize and detect G4s with a very low detection limit. In contrast to another approach that make use of flexible alkylammonium groups (Hong et al., 2010; Zhang et al., 2015), we have selected imidazolium cationic groups directly connected to the TPE core (Scheme 1), as these were found to provide high affinity toward human telomeric G4s, through the combination of electrostatic interactions with the DNA backbone and π-type interactions with the nucleobases of the G4 loops (Rubio-Magnieto et al., 2015).

In this paper, we report on the synthesis and the optical properties of a novel compound **TPE-Im**, see Scheme 1. We study its G4 binding properties with human telomeric DNA (HT-DNA) sequences, by means of circular dichroism (CD), and fluorescence spectroscopies, and isothermal titration calorimetry (ITC). We have selected the (HT-DNA) sequences **Tel22** (5′-AGG GTT AGG GTT AGG GTT AGG G-3′) because of their relevance as G4 targets in cancer research, and because these sequences can adopt different intramolecular G-quadruplex structures depending on the solution conditions.

## Materials and Methods

### Materials

Dry DMF and THF were obtained by a solvent purification system PureSolve MD5 from Innovative Technology. Preparative purifications were performed by silica gel flash column chromatography (Merck 40–60 mm). Solvents used as eluents are technical grade.

### Synthesis

The synthesis of 1,1,2,2-tetrakis(4-bromophenyl)ethane (**TPE-Br**) was performed according to literature procedures (Schultz et al., 2003).

### Synthesis of Tetra-Imidazole-Appended Tetrakis(*p*-phenylene)Ethylene (TIPE)

1,1,2,2-tetrakis(4-bromophenyl)ethene (0.500 g, 0,772 mmol), imidazole (0.42 g, 6.177 mmol; 8 eq.), potassium carbonate (0.750 g, 5.435 mmol; 7 eq.) and CuSO_4_ (0.025 g, 0.157 mmol; 0.2 eq.) were placed into a Schlenk flask under an argon atmosphere and anhydrous DMF (2 mL) was added. The solid mixture was then heated to 140°C for 24 h. The reaction mixture was cooled to room temperature and was washed three times (3 × 20 mL) with distilled water. The remaining solid residue was extracted with methanol (3 × 10 mL) and the methanol solution was filtered and evaporated under vacuum. The crude product was then purified by column chromatography on silica gel eluting with a gradient of dichloromethane:methanol (98:2–90:10) to give **TIPE** as a colorless solid. Yield: 0.220 g (0.370 mmol, 48%). Spectroscopic data for **TIPE** are the same as previously described in the literature (Sinha et al., 2017). ^1^H NMR (300 MHz, DMSO-d_6_): δ = 8.27 (s, 4H), 7.75 (s, 4H), 7.54 (d, ^3^*J*_H−H_ = 8.5 Hz, 8H), 7.19 (d, ^3^*J*_H−H_ = 8.5 Hz, 8H), 7.09 ppm (br. s, 4H) ppm.

### Synthesis of TPE-Im

In a 50 mL Schlenk tube under an argon atmosphere, **TIPE** (0.100 g, 0.168 mmol) was dissolved in anhydrous DMF (15 mL). Iodomethane (5 mL, 0.800 mmol; 470 eq.) was added at once. The reaction mixture was stirred at room temperature for 72 h. **TPE-Im** is then precipitated in diethyl ether (400 mL) and filtered leading to a yellow solid. Yield: 0.180 g (0.146 mmol, 87%). ^1^H NMR (300 MHz, CD_3_OD) δ = 9.55 (s, 4H), 8.05 (t, *J*_H−H_ = 1.7 Hz, 4H), 7.76 (t, *J*_H−H_ = 1.8 Hz, 4H), 7.61 (d, ^3^*J*_H−H_ = 8.7 Hz, 8H), 7.42 (d, ^3^*J*_H−H_ = 8.7 Hz, 8H), 4.03 (s, 12H, N-CH_3_) ppm. ^13^C{^1^H} NMR (101 MHz, CD_3_OD) δ = 143.8, 142.2, 135.3, 134.2, 125.7, 123.2, 122.6, 37.0 ppm. HR-MS (ESI-TOF^+^): *m/z* calculated C_42_H_40_${\text{N}}_{8}^{4+}$ 164.0844 [M – 4I]^4+^, found 164.0845. UV-vis (H_2_O) λ (ε L. mol^−1^. cm^−1^) = 260 (29 800), 293 (20 800), 322 (16 800) nm.

### UV-Vis Absorption and Circular Dichroism Spectroscopy

UV-Vis absorption spectra of pure **TPE-Im** were recorded at 25°C on a JASCO V-750 spectrophotometer in 10 mm quartz cells (Hellma). The extinction coefficients were determined by preparing solutions of **TPE-Im** at different concentration, and the concentration range was judiciously chosen to remain in the linear range of the Beer-Lambert relationship (A ~ 0.2–0.8). For **TPE-Im**/**G4** mixtures, UV-Vis absorption and Circular Dichroism (CD) measurements were recorded using a ChirascanTM Plus CD Spectrometer from Applied Photophysics. The measurements were carried out using 2 mm suprasil quartz cells from Hellma Analytics. The spectra were recorded at 20°C between 225 and 600 nm, with a bandwidth of 1 nm, time per point 1 s. The buffer water solvent was Tris-EDTA prepared from 1 M Tris-Cl and 0.5 M EDTA to achieve a 10 mM Tris-Cl and 1 mM EDTA final buffer at pH 7.5. The buffered water solvent reference spectra were used as baselines and were automatically subtracted from the CD and UV-Vis absorption of the samples. All the spectra were treated by using OriginPro 2018 software.

### Fluorescence Spectroscopy

Emission spectra of pure **TPE-Im** were recorded at 25°C on a fluorescence spectrophotometer (FS920, Edinburgh Instruments) equipped with a calibrated photomultiplier in a Peltier (air cooled) housing (R928P, Hamamatsu), with a 450 W continuous xenon arc lamp as the excitation source for steady-state photoluminescence measurements using a quartz cuvette with 1.0 cm excitation path length. Emission spectra of **TPE-Im**/**G4** mixtures were recorded using a ChirascanTM Plus CD spectrophotometer from Applied Photophysics equipped for fluorescence measurements. The measurements were carried out using 4 mm by 10 mm suprasil quartz cells from Hellma Analytics. The spectra were recorded at 20°C between 270 and 700 nm, with a bandwidth of 2.0 nm, time per point 0.5 s. The buffer water solvent was Tris-EDTA at 10 mM Tris-Cl and 1 mM EDTA, pH 7.5, same as CD and UV-vis experiment. All the spectra were treated by using OriginPro 2018 software.

### Molecular Docking

**TPE-Im** was built within the Avogadro molecular editor (Hanwell et al., 2012). Molecular mechanics calculations were then performed to optimize the geometry of the **TPE-Im** molecule. For this, a two-step minimization procedure, i.e., a steepest descent optimization followed by a conjugate gradient optimization, was performed with the General Amber Force Field (GAFF) (Wang et al., 2004). The energy convergence criterion was set at 10^−5^ kJ. mol^−1^ for the energy minimization. The coordinates of the two G-quadruplexes (G4) were obtained from the Protein Data Bank (PDB ID: 143d and PDB ID: 2hy9). For each G4, we extracted the different NMR conformations to perform ensemble docking calculations, i.e., six conformations for the 143D target and 10 conformations for the 2HY9 target. Docking calculations were performed with the AutoDock Vina package (Trott and Olson, 2009). As we have no *a priori* knowledge of the **TPE-Im** binding mode along the G4 structures, a sufficiently large grid was built around each G4 structure to allow the exploration of the entire G4 surface during the docking calculations. A large grid size of 100 × 100 × 100 Å^3^ with a spacing of 0.375 Å was thus considered. The center of the grid box was located on the center-of-mass of the G4 targets. As the grid presents an important size, an exhaustiveness value of 32 was chosen, a larger one than the default value, i.e., eight (Jaghoori et al., 2016). **TPE-Im** was set as a flexible entity, and we considered various torsions between imidazolium and benzyl moieties. The 10 most energetically favorable complexes were retained for each docking calculation. The PyMOL molecular visualization system was used to depict illustrations of the **TPE-Im** docking calculations (Delano, 2002).

### Fluorescence Resonance Energy Transfer (FRET) Melting Assays

FRET melting assays were performed according to Decian et al. (2007), Renciuk et al. (2012), and Rubio-Magnieto et al. (2015), using a synthetic double-dye labeled oligonucleotide called **F21T** 5′-FAM-GGG(T2AG3)3-TAMRA-3′ (purchased with the highest purity grade from Eurogentec, Belgium). The solutions were prepared at a concentration of around 0.3 μM (ODN concentration) in 10 mM lithium cacodylate buffer (pH = 7.2) in presence of 100 mM KCl. The solutions were first heated to 90°C for 315 min in the corresponding buffer conditions and then slowly cooled down at 1.5°C/min to 20°C to support the formation of G4 secondary structure. The mixtures were equilibrated at 25°C during 5 min. The FRET spectra were measured using a ChirascanTM Plus CD Spectrophotometer equipped for fluorescence measurements. The samples were excited at 492 nm and the fluorescence emission spectra were collected between 500 and 700 nm. The temperature was varied from 20 to 95°C at a rate of 0.3°C/min. The melting of the **F21T** was monitored by measuring the fluorescence of FAM (at 516 nm), as described in reference (Decian et al., 2007; Renciuk et al., 2012; Rubio-Magnieto et al., 2015). The FAM emission intensity was normalized and ΔT_1/2_ was defined as the temperature for which the normalized emission equals 0.5. For the selectivity studies, a solution of 10 molar equivalents of a dsDNA competitor (~3 μM in double-strand) was added into the **F21T/TPE-Im** solution and the final solution was equilibrated at 25°C during 5 min. The dsDNA competitor (**ds43**) is a 43 base pairs with sequence: 5′-CGT CAC GTA AAT CGG TTA ACA AAT GGC TTT CGA AGC TAG CTT C-3′, hybridized with its complementary sequence. All the spectra were treated by using OriginPro 2018 software.

### Isothermal Titration Calorimetry

Isothermal titration calorimetry (ITC) experiments were carried out at 20°C on a Microcal ITC200 instrument (Microcal). The titration cell was filled with a solution of 10–20 μM **Tel22** DNA and the syringe was loaded with a **TPE-Im** solution of 600–2,000 μM. Experiments consisted of a series of 26 injections of ligand from rotating syringe (speed 750 rpm) into the thermostatic cell (initial delay of 60 s, duration of 2 s and spacing of 120 s). Control experiments were carried out where the ligand solution was added into the buffer containing cell. The corrected ITC titrations were treated by using Origin 7.0 software.

## Results and Discussion

### Synthesis

**Scheme 2:**
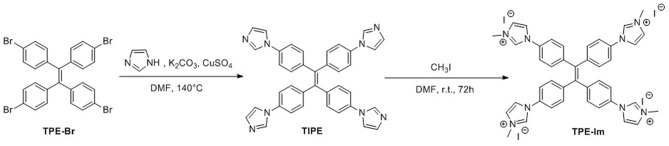
Synthesis of **TPE-Im**.

TPE-bridged tetraimidazolium salt (**TPE-Im**) was synthesized in two steps starting from 1,1,2,2-tetrakis(4-bromophenyl)ethene (**TPE-Br**) by using a slightly modified version of a previously reported procedure (Scheme 2) (Sinha et al., 2017). First, the imidazole was linked to the TPE core through Ulmann coupling reaction leading to **TIPE** in 47% yield (Supplementary Figure 1). Then, the alkylation of **TIPE** with an excess of iodomethane afforded the tetraimidazolium salt in a quantitative yield (Supplementary Figures 2, 3). The ^1^H NMR spectrum of **TPE-Im** in CD_3_OD clearly shows the downfield signal of the acidic proton (C-H) of the imidazolium ring at δ = 9.55 ppm and the signal of N-CH_3_ at δ = 4.03 ppm (see Supplementary Figure 2). The molecular mass peak of **TPE-Im** was observed by using ESI-TOF mass spectrometry at *m/z* = 164.0845, as expected for this tetracationic species (calculated *m/z* = 164.0844 [M−4I]^4+^).

### Optical Properties

**Figure 1:**
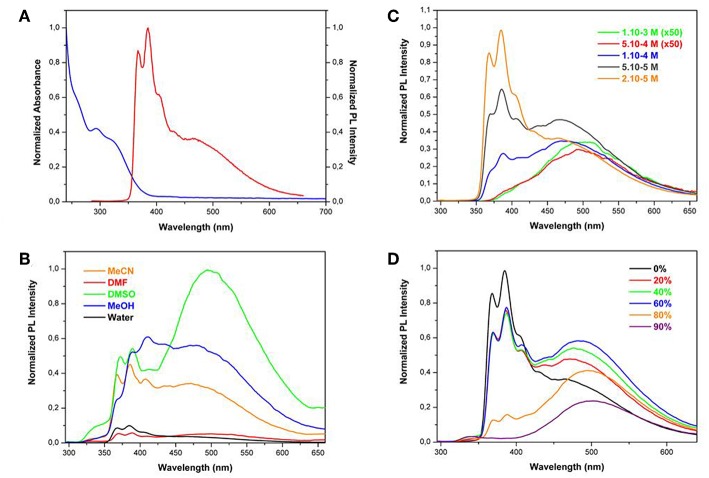
**(A)** UV-Visible absorption (blue) and PL spectra (red) of **TPE-Im** in water (0.8 % DMSO) at 2 × 10^−5^ M (λ_exc_ = 265 nm). **(B)** PL spectra of **TPE-Im** in water (black), methanol (blue), acetonitrile (orange), DMF (red), and DMSO (green) at 2 × 10^−5^ M (λ_exc_ = 265 nm). **(C)** PL spectra of **TPE-Im** at different concentrations (λ_exc_ = 265 nm). **(D)** PL spectra of **TPE-Im** in water/THF mixtures with different THF fractions (Concentration: 2 × 10^−5^ M, λ_exc_ = 265 nm).

The optical properties of **TPE-Im** were studied by UV-Vis absorption and photoluminescence (PL) spectroscopies. The absorption and emission spectra of **TPE-Im** in water (0.8 % DMSO) are shown in Figure 1A. Two unstructured absorption bands are observed at 293 nm and 322 nm, which could be assigned to π-π^*^ transition (Salimimarand et al., 2017; Kayal et al., 2018). In water, **TPE-Im** shows two emission bands: a structured one at ~380 nm and another broader and weaker at ~480 nm. The shape of the emission spectra, especially the ratio of the intensity of the two bands at ~380 nm and ~480 nm, is dependent on the nature of the solvent. Figure 1B shows the emission spectra of **TPE-Im** in polar aprotic (acetonitrile, DMF, DMSO) and protic (MeOH) solvents. A strong increase of the emission band at ~480 nm in the emission spectra of **TPE-Im** in pure DMSO is noticed compared to water (0.8% DMSO). To understand the nature of the band at longer wavelength, the effect of concentration on the emission profile of pure **TPE-Im** in water is studied (from 10^−5^ to 10^−3^ M, see Figure 1C). At high concentration (5 × 10^−4^–10^−3^ M), only the broad emission band at longer wavelength remains but its intensity gradually decreases. The solvatochromic and concentration effects may indicate that the broad peak at around 470 nm is due to a twisted intramolecular charge transfer (TICT) state, as previously observed for tetrapyridinium-based TPE (Grabowski et al., 2003; Shigeta et al., 2012). To investigate the potential AIE behavior of **TPE-Im**, emission spectra were then recorded in water/THF mixtures with different THF fractions in view of fine-tuning the THF content as well as the aggregation extent (Figure 1D). Adding a poorer solvent (THF) to the water solution results in the gradual disappearance of the broad emission band around 380 nm. Only the broad emission band around 480 nm remains at high THF content (90%). However, adding THF to a solution of **TPE-Im** in water not only leads to a modification of the emission profile but also to an important decrease of the emission intensity. These results clearly indicate that **TPE-Im** is AIE-inactive in THF/water solution conditions.

**Figure 2:**
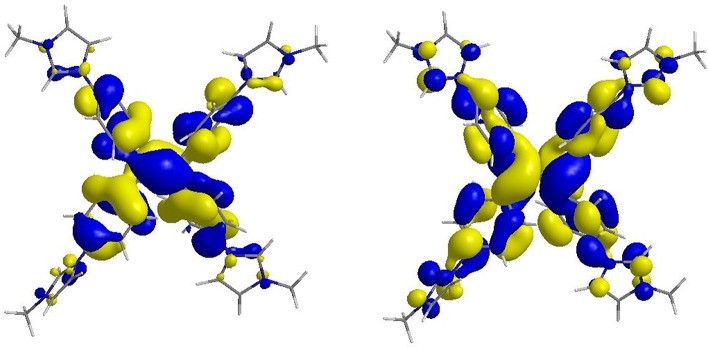
Isosurface plots of frontier orbitals in the ground state: HOMO (left) and LUMO (right) orbitals of **TPE-Im**.

DFT B3LYP 6/31G^*^ calculations have been carried out on **TPE**-**Im** to both get information about its optimized structure and its frontier orbitals plots (Frisch et al., 2009). As observed with other TPE derivatives, the optimized structure shows a four-winged propeller-like conformation with slightly different torsion angles between the ethylene core and the adjacent phenyl rings in the range of what is usually measured from the crystals (φ_1_ = 52.0°, φ_2_ = 52.9°, φ_3_ = 50.1°, φ_4_ = 53.8°, φ_av_ = 52.2°) (Supplementary Figure 4 and Supplementary Table 1) (Cai et al., 2018; Zhang et al., 2019). Examination of the frontier orbital plots indicates that HOMO and LUMO orbitals are mainly located on the TPE core with a relatively low contribution of the imidazolium rings, which suggests that the photophysical properties mainly arise from the TPE core (Figure 2).

### Binding to Human Telomeric Sequence

**Figure 3:**
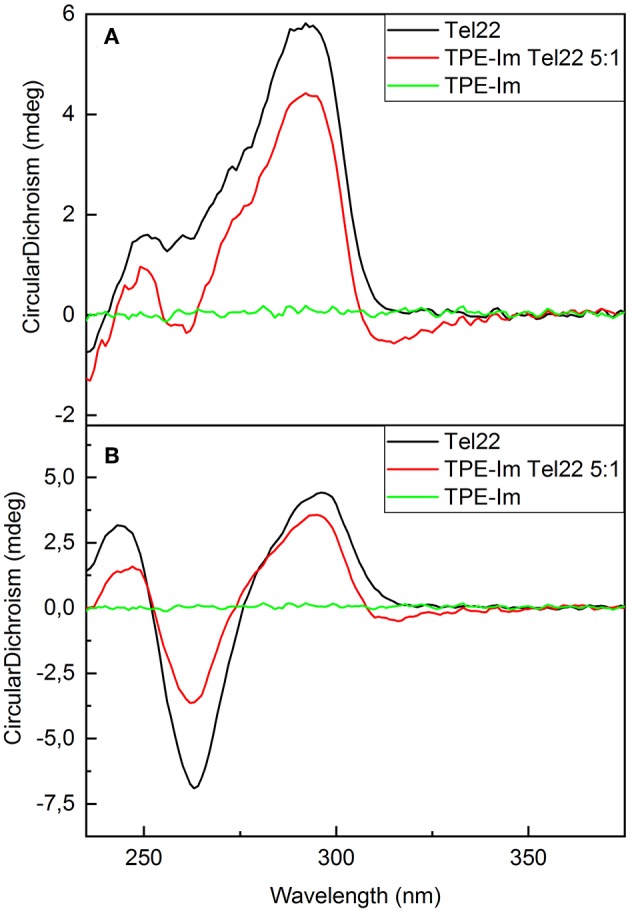
CD spectra of **(A) TPE-Im:Tel22** in TE buffer + 100 mM KCl; B) **TPE-Im:Tel22** in TE buffer + 100 mM NaCl. The molar ratio for **(A)** and **(B)** is 5:1 in **TPE-Im:Tel22**.

The human telomeric sequence studied here was **Tel22** of d[AG_3_(T_2_AG_3_)] sequence, which presents different G4 loop structures depending on the nature of the alkali cation added in solution. With Na^+^ at 100 mM, **Tel22** specifically folds into G4 parallel loop structure. In contrast, with K^+^ at 100 mM, it folds into a mixture of parallel and anti-parallel G4 conformation, in a dynamic equilibrium between hybrid structures (Dai et al., 2008; Phan, 2010). CD spectra of **TPE-Im** in interaction with different **Tel22** in different aqueous solution media are presented in Figure 3. The CD spectra of **Tel22** in presence of KCl show a positive peak at 290 nm and a shoulder at 250 nm, which is typical of **Tel22** with K^+^ (Rubio-Magnieto et al., 2015). In the **TPE-Im**/**Tel22** mixture (5:1), the CD bands related to the G-quadruplex are barely modified, with a slight decrease of the signal intensity. A weak negative peak appears at 315 nm, in the spectral range where only **TPE-Im** absorbs. Indeed, pure **TPE-Im** in aqueous solution does not show any CD signal. CD spectra of **Tel22** in aqueous solution with NaCl show specific antiparallel G-quadruplex structure, with a positive peak at 295 nm and strong negative peak at 265 nm (Wang and Patel, 1993; Rubio-Magnieto et al., 2015). These signals are reduced in presence of **TPE-Im**, and a very weak negative signal at 315 nm is present. As the concentration of **Tel22** is maintained constant for both experiments (see Supplementary Figure 5), the modifications in the spectra seem to be directly linked to the interaction between **TPE-Im** and **Tel22**.

### Molecular Modeling of TPE/G4 Binding Modes

**Figure 4:**
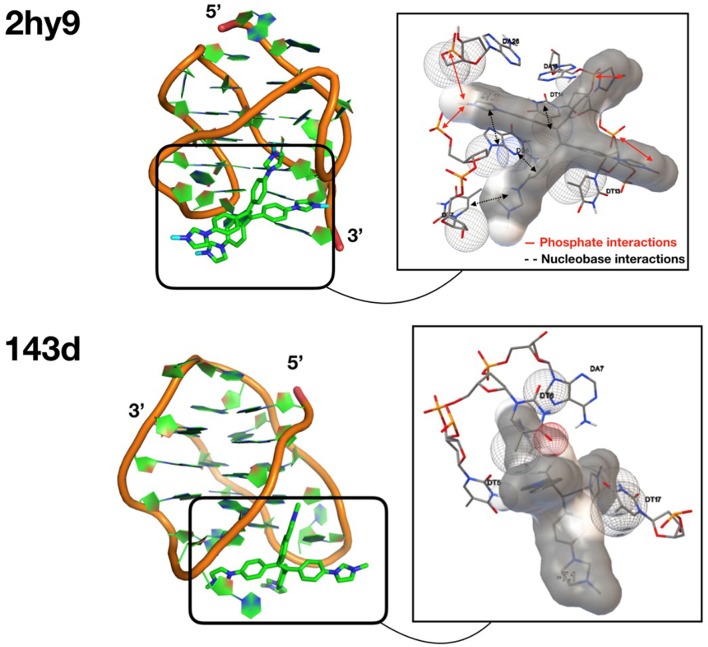
Structures of the most stable docking solutions for each G4 target (see PDB ID on the left). The close contacts are shown on the right, with **TPE-Im** surrounded by its van der Waals surface. The spheres in dots represent van der Waals spheres of the atoms of G4 in contact with the **TPE-Im** ligand.

To gain insights into the affinity and the binding modes of **TPE-Im** with **Tel22**, we perform docking calculations using the AutoDock Vina package (Trott and Olson, 2009), see computational details in the Supporting Information. We consider several conformations for both G4 targets (six conformations for the PDB IDs 143D with Na^+^, and 10 conformations for 2HY9 with K^+^) to take into account, in an implicit manner, the flexibility of the targets. The affinity, calculated on the 10 most stable docking solutions for each conformation of both G4 targets, are reported in Supplementary Figure 10. In Supplementary Table 2, we report the statistical analysis of the related docking calculations. For the G4 target in K^+^ (2HY9), the best average affinity is found for the third conformation (model 3 in the NMR structure), with the global maximal affinity (lowest binding energy) of E_*b*_ = −7.3 kcal/mol (corresponding to the structure shown in Figure 4 top). Let us note that the average RMSD of the 10 recorded docking solutions is also the lowest for the third conformation, which emphasizes a stable binding mode. For the G4 target in Na^+^ (143D), the most stable set of docking solutions is found for the conformation #5 (E_*b*_ = −7.1 kcal/mol, Supplementary Figure 10 and Supplementary Table 2), see its structure in Figure 4 bottom.

The most energetically favorable docking solutions for **TPE-Im**/**Tel22** complex present similar calculated binding energies [E_*b*_ = −7.3 kcal/mol for the **TPE-Im**/G4(2HY9) complex and E_*b*_ = −7.1 kcal/mol for the **TPE-Im**/G4(143D)]. However, the superpositions of the 10 most stable docking solutions (Supplementary Figures 11, 12) depict different **TPE-Im/**G4 binding modes depending on the alkali cation. For the 2HY9 G4, docking solutions are well-superposed, indicating one clear binding mode of **TPE-Im** (Supplementary Figure 11). Nine of the ten **TPE-Im**/G4 (2HY9) docking solutions are grouped into only two clusters (Supplementary Figure 11). Moreover, these two clusters are similar between them regarding the weak RMSD value with respect to the most stable docking solution (Supplementary Table 3). Therefore, docking calculations show that **TPE-Im** ligand has a single, well-defined binding mode, as shown in Figure 4 with **TPE-Im** buried at the bottom of the 2HY9 target. The close contacts of the most stable docking solution show that the charged nitrogen atoms of **TPE-Im** ligand are close to the phosphate groups of 2HY9. A proximity between aromatic nucleobases and aromatic ligand moieties is also observed. The affinity of the **TPE-Im** ligand to the G4 target (2HY9) arises from concomitant electrostatic interactions and stacking interactions.

The possible binding modes are different for the second G4 target (PDB ID: 143D). Although the **TPE-Im** is also bound in a wide groove of 143D, the 10 docking solutions are clearly much less superimposed than for 2HY9, illustrating various binding modes. The docking solutions are therefore grouped into clusters (Supplementary Figure 12), with high differences in RMSD values (Supplementary Table 4) compared to the case of **TPE-Im**/G4(2HY9) complex. The close contacts of the most stable docking mode do not involve phosphate groups (see Figure 4 bottom), and the aromatic moieties of the **TPE-Im** are not in contact with nucleobases, with a quasi-perpendicular binding mode between the G4(143D) tetrad and the main plane of **TPE-Im**. Such a binding mode explains why the affinity of the **TPE-Im** ligand with the 143D target is lower compared to the 2HY9 target. Let us note that the most stable docking solution of **TPE-Im/**G4(143D) implies a particular conformation of the G4(143D) target, i.e., an opening of the G4 structure through disruption of some interactions between nucleobases. The docking calculations do not take into account the entropic effects, but such a binding mode could have a high entropic cost, needing an opening of 143D. These docking calculations indicate a groove-binding mechanism of **TPE-Im** to HT-G4, with more diverse possible binding modes for 2HY9 G4 compared to 143D G4.

### Fluorescence Spectroscopy of TPE/G4

**Figure 5:**
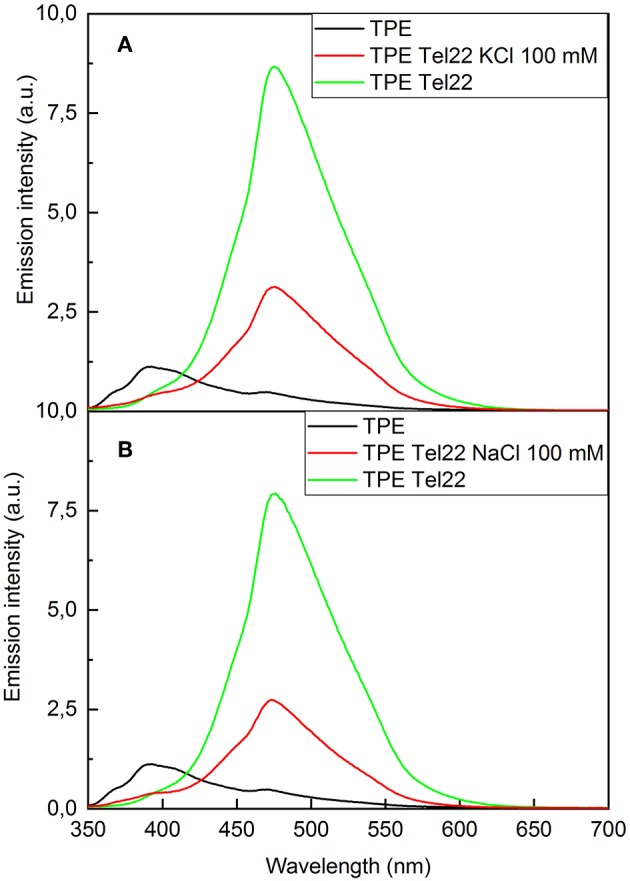
Fluorescence spectra of **(A) TPE-Im:Tel22** in TE buffer (λ_exc_ = 265 nm). **(B) TPE-Im:Tel22** in TE buffer (λ_exc_ = 265 nm). The molar ratio for **(A)** and **(B)** is 5:1 in **TPE-Im:Tel22**.

The interactions between **TPE-Im** and **Tel22** have also been studied by fluorescence spectroscopy, as shown in Figure 5. Pure **TPE-Im** in TE buffer (black lines Figure 5) reaches an emission maximum at 390 nm, with a broad shoulder at 470 nm. In the presence of salt or in physiological environment buffer, only minor changes are observed in the emission profile compared to pure **TPE-Im**: the intensity of the emission band at 390 nm is slightly modified whereas the emission band at 470 nm shows the same intensity with or without KCl in the solution (Supplementary Figures 6, 7). Adding **Tel22** to a solution containing NaCl, the emission peak at 470 nm of **TPE-Im** strongly increases while the peak at 390 nm vanishes. This increase of fluorescence for **TPE-Im** may be due to the concomitant electrostatic interactions and stacking interactions taking place with G4 (see above), which leads to restricted intramolecular rotations and thus, an exalted emission band around 480 nm; a typical value for TPE derivatives in aggregated state (Huang et al., 2012; Odabas et al., 2013; Sinha et al., 2017). The same behavior is observed with **Tel22** in an aqueous solution containing KCl, the shoulder at 390 nm decreases while the peak at 470 nm strongly increases, with about the same intensity than with NaCl. This “light-up” fluorescence effect is influenced by the presence of added salt in the mixture. Indeed, the increase of the fluorescence is significantly higher when no salt is added (note that pure **Tel22** form G-quadruplex structures even without adding salt to the solution, see Supplementary Figure 8), while it is lower with a concentration of added salt at 100 mM of NaCl or KCl (see green lines vs. red lines in Figure 5, respectively). It is likely that G4 binding and further aggregation of **TPE-Im** over G4 is more favorable when the concentration of alkali cations is low, with a lesser extent of screening by alkali ions than for solutions with 100 mM of added salt. The selectivity of **TPE-Im** toward double stranded DNA (**dsR**_**20**_) vs. G4 DNA (**Tel22**) was estimated through fluorescence measurements. Adding **Tel22** to **TPE-Im** results in a fluorescence exaltation three or five times higher than that with double-stranded DNA (see Supplementary Figure 9).

### Stability and Specificity of TPE/G4 Interaction

**Figure 6:**
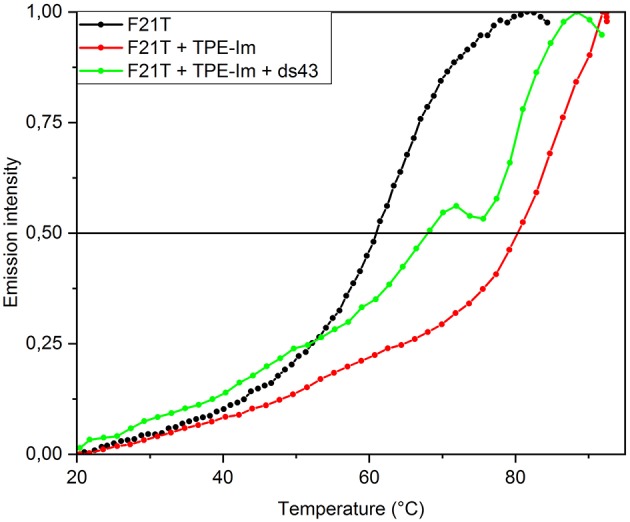
FRET melting essay for solutions of pure **F21T** (200 nM), and in the presence of 1 μM **TPE-Im** with or without 10 equivalent of competitor double-stranded DNA (ds43). The emission intensity corresponds to normalized FAM fluorescence (λ_exc_ = 492 nm). All measurements were performed in a 10 mM lithium cacodylate buffer (pH = 7.2) with 100 mM KCl.

Fluorescence Resonance Energy Transfer (FRET) melting assays were performed in order to assess the stability and selectivity of **TPE-Im** for G-quadruplex over double-stranded DNA (dsDNA). These experiments are based on the measurement of the melting temperature of double-dye labeled G4, by monitoring the FRET between two dyes located at 5′ and 3′ positions when the G4 goes from folded to unfolded states upon increasing the temperature of the solution. The oligonucleotide used here is a modified **Tel22** sequence end-capped with a fluorescein amidite dye (FAM) at 5′-end and a tetramethylrhodamine (TAMRA) at 3′-end, the labeled oligonucleotide FAM-5′-GGG(T2AG3)3-TAMRA-3′, referred to as **F21T**. This oligonucleotide has been widely studied in the literature for assessing stabilization of G4 and selectivity by ligands (Decian et al., 2007; Renciuk et al., 2012). After a preliminary heating/cooling cycle of **F21T** to make sure of its folding into a G4, **TPE-Im** is added and the emission of the donor dye (i.e., FAM, λ_exc_ = 492 nm) has been followed as a function of temperature, as it has already been done by us and others. This method has been shown to give more reproducible results than the sensitized emission of the acceptor (i.e., TAMRA). The G4 structure unfolds when the temperature increases and thereby the light emission of the donor (i.e., FAM) increases. The denaturation of G4 has been followed for pure **F21T**, for a mixture **F21T/TPE-Im** in a 1:5 molar ratio, and for a mixture **F21T/TPE-Im/ds43** in a 1:5:10 molar ratio in K^+^ buffer conditions, as shown in Figure 6. The determination of the half-melting temperature difference (ΔT_1/2_) between the pure **F21T** and **F21T/TPE-Im** is a quantitative measurement of the stabilization effect due to the added ligand. The results show T_1/2_ of 61.0 and 80.5°C for the pure **F21T** and **TPE-Im**/**F21T**, respectively. These results show a strong stabilization of G-quadruplex by **TPE-Im**, with a ΔT_1/2_ = 19.5°C. The addition of 10 equivalents of dsDNA competitor (**ds43**) disturbs the **TPE-Im/F21T** stabilization, with a T_1/2_ of 68°C (ΔT_1/2_ = 7°C), despite the high content in competitor. This means that **TPE-Im** is more specific to **Tel22** G4 DNA conformation than to dsDNA conformation, even with 10 times the concentration of double-stranded DNA vs. G4 DNA. The **TPE-Im/F21T** complex seems disturbed by the high concentration on competitive double stranded DNA but we cannot see a complete drawback to pure **F21T** T_1/2_. In comparison with the previous work of our group, the **TPE-Im/F21T** complex is less disrupted than the porphyrin's one (Rubio-Magnieto et al., 2015). It is likely due to its geometry that is particularly adapted to the G4 structure (see above). Note that we observe a jump in the green curve at 72°C which may be due to a partial intercalation of **TPE-Im** in the unwound double stranded DNA (that has a melting temperature slightly under at 67°C).

### Isothermal Titration Calorimetry

**Figure 7:**
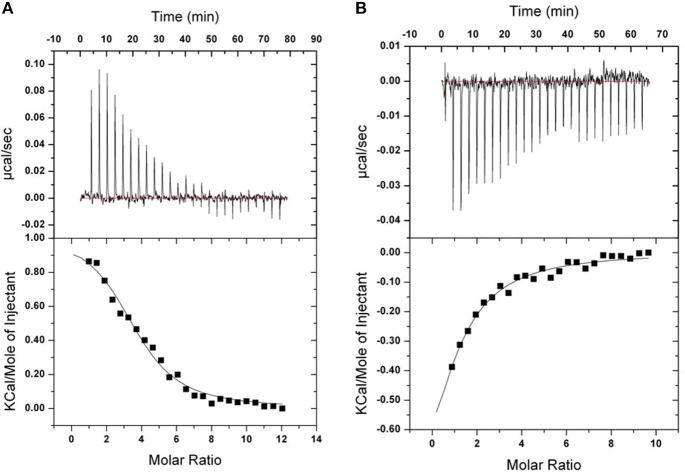
ITC binding isotherms obtained at 20°C for **TPE-Im** in two different buffers. **(A)** 10 mM Tris, 1 mM EDTA, 100 mM KCl, pH 7.4. **(B)** 10 mM Tris, 1 mM EDTA, 100 mM NaCl, pH 7.4. The upper isotherms indicate **Tel22** DNA binding raw data. The lower curves are obtained after integration of individual heat flow signals as function of DNA/ligand molar ratio in the calorimeter cell.

Isothermal titration calorimetry experiments were carried out to quantify the binding of **TPE-Im** to **Tel22** DNA in presence of NaCl or KCl, see Figure 7 and Table 1. In both cases, binding seems to be strongly driven by favorable entropy changes to a much greater extent than with the flexible cationic TPE-based ligands previously described (Hong et al., 2010). This different behavior is possibly due to the dehydration of the larger aromatic surface of **TPE-Im** whereas entropic penalty occurs upon binding for the TPE-derivatives having flexible side-chains. While a small favorable enthalpy change is observed in the presence of NaCl, an endothermic binding occurs in the presence of KCl. It is possible that this small difference is due to the better pre-organization of **Tel22** in a single G4 parallel loop structure with NaCl and in a mixture of parallel and anti-parallel G4 conformation with KCl, but we interpret this with caution because of the small difference of measured association constants. Overall, the observed association constants reach 3.2 ± 1.1 10^5^ M^−1^ in NaCl and 2.4 ± 1.0 10^5^ M^−1^ in KCl (Figure 7), which are typical of TPE-based G-quadruplex ligands (Hong et al., 2010).

**Table 1:** Estimates of the association constants K_*a*_ and thermodynamic parameters, as determined by ITC.

	**TPE-Im/HT-G4**	
	**in NaCl**	**in KCl**
*K_*a*_* (M^−1^)	3.2 (±1.1).10^5^	2.4 (±1.0).10^5^
ΔH (kcal.mol^−1^)	−0.3 (±0.06)	0.8 (±0.1)
–TΔS (kcal.mol^−1^.K^−1^)	−7.1 (±0.2)	−8 (±0.2)

## Conclusions

We have reported on the design of a novel TPE-based fluorophore that features methyl-imidazolium groups directly tethered to the TPE core, **TPE-Im**. We found that this original design endows peculiar optical properties and a significant shift from 380 to 480 nm in the fluorescence emission that is triggered by aggregation. Binding to a (HT-DNA) sequences **Tel22** was characterized by CD spectroscopy without and with salts (NaCl, KCl), and molecular modeling suggest a predominant *side-on* groove binding, with an opening of the grooves in the case of **Tel22** in KCl. Fluorescence spectroscopy demonstrates that the probe **TPE-Im** shows a turn-on fluorescence emission upon binding to **Tel22**, and interestingly, also displays a significant shift in fluorescence emission (380 to 480 nm). A FRET melting assay shows a strong stabilizing effects of **TPE-Im** on the secondary folded structure of **Tel22** (ΔT_1/2_ = 19.5°C), with a good degree of selectivity to G4 against double-stranded DNA. Finally, ITC was used to determine the association constants, which are in the range 2.4–3.2 × 10^5^ depending on the solution conditions. Overall, we believe these results set the stage for the further use of **TPE-Im** as a novel probe that display turn-on and ratiometric responses upon binding to DNA G-quadruplexes.

## Data Availability

The raw data supporting the conclusions of this manuscript will be made available by the authors, without undue reservation, to any qualified researcher.

## Author Contributions

All authors listed have made a substantial, direct and intellectual contribution to the work, and approved it for publication.

### Conflict of Interest Statement

The authors declare that the research was conducted in the absence of any commercial or financial relationships that could be construed as a potential conflict of interest.
