# Supplementary material for: A Cationic Tetraphenylethene as a Light-Up Supramolecular Probe for DNA G-Quadruplexes
Source: Front Chem. 2019 Jul 11;7:493. doi: 10.3389/fchem.2019.00493 (PMC6637260; doi:10.3389/fchem.2019.00493)
Supplement: Supplementary file 1 [file Data_Sheet_1.docx]

Supplementary Material

A cationic tetraphenylethene as a light-up supramolecular probe for DNA G-quadruplexes

Clément Kotras,^1,2^ Mathieu Fossépré,^2^ Maxime Roger,^1^ Virginie Gervais,^3^ Sébastien Richeter,^1^ Philippe Gerbier,^1^ Sébastien Ulrich,^4^ Mathieu Surin,^2^* Sébastien Clément^1^*

^1^ ICGM Institut Charles Gerhardt Montpellier, UMR 5253, CNRS, Université de Montpellier, ENSCM, 34095 Montpellier, France.

^2^ Laboratory for Chemistry of Novel Materials, Center of Innovation and Research in Materials and Polymers (CIRMAP), University of Mons-UMONS, 7000 Mons, Belgium

^3^ Institut de Pharmacologie et de Biologie Structurale, CNRS, IPBS, Université de Toulouse, 31077 Toulouse, France

^4^ Glycochimie et Reconnaissance Moléculaire, Institut des Biomolécules Max Mousseron, IBMM, UMR 5247, CNRS, Université de Montpellier, ENSCM, 34095 Montpellier, France

*** Correspondence:**Pr. Mathieu Surin and Pr. Sébastien Clément
mathieu.surin@umons.ac.be; sebastien.clement1@umontpellier.fr

**Table of contents**

**Supplementary Figure 1**. ^1^H NMR spectrum of **TIPE** in DMSO-*d*_6_. 3

**Supplementary Figure 2**. ^1^H NMR spectrum of compound **TPE-Im** in CD_3_OD 3

**Supplementary Figure 3**. ^13^C{^1^H} NMR spectrum of compound **TPE-Im** in CD_3_OD. 4

**Supplementary Figure 4**. DFT optimized structure of TPE-Im. 4

**Supplementary Table 1.** Selected torsion angle value found in TPE-Im optimized structure. Indications between brackets correspond to the torsion angle labelling in the article. 5

**Supplementary Figure 5.** UV-visible absorption spectra of A) **TPE-Im:Tel22** in TE buffer + 100mM KCl; B) **TPE-Im:Tel22** in TE buffer + 100mM NaCl. The molar ratio for A) and B) is 5:1 in **TPE-Im:Tel22.** 5

**Supplementary Figure 6.** Emission spectra of **TPE-Im** (λ_exc_ = 265 nm) at different concentrations with (black and green curve) or without KCl (red and blue curve) in comparison with the fluorescence of **TPE-Im** in the presence of **Tel22** and KCl at 5:1 ratio (pink curve). 6

**Supplementary Figure 7.** Emission spectra of **TPE-Im** (λ_exc_ = 265 nm) at 2 μM in a in physiological environment buffer. 6

**Supplementary Figure 8**. CD Spectra of **Tel22** at 3µM in TE buffer. 7

**Supplementary Figure 9.** Emission spectra of **TPE-Im** (λ_exc_ = 265 nm) in the presence of human telomeric DNA sequence (**Tel22**) or double stranded DNA (**dsR_20_**) at different molar ratio (1:1 TPE-DNA in black and green; 5:1 TPE-DNA in red and blue). 7

**Supplementary Figure 10.** Affinity scores of the 10 recorded docking solutions for each conformation of G4(2HY9) (left) and G4(143D) (right) 8

**Supplementary Table 2.** Statistical analysis of the recorded docking calculations 8

**Supplementary Figure 11.** Docking solutions of the **TPE-Im**/G4(2HY9, conformation 3) 10

**Supplementary Table 3.** Affinity score and RMSD of the 10 docking solutions between **TPE-Im** and G4(2hy9, conformation 3) 11

**Supplementary Figure 12.** Docking solutions of the **TPE-Im**/G4(143D, conformation 5) 12

**Supplementary Table 4.** Affinity score and RMSD of the 10 docking solutions between **TPE-Im** and G4(143D, conformation 5) 13

**References** 14


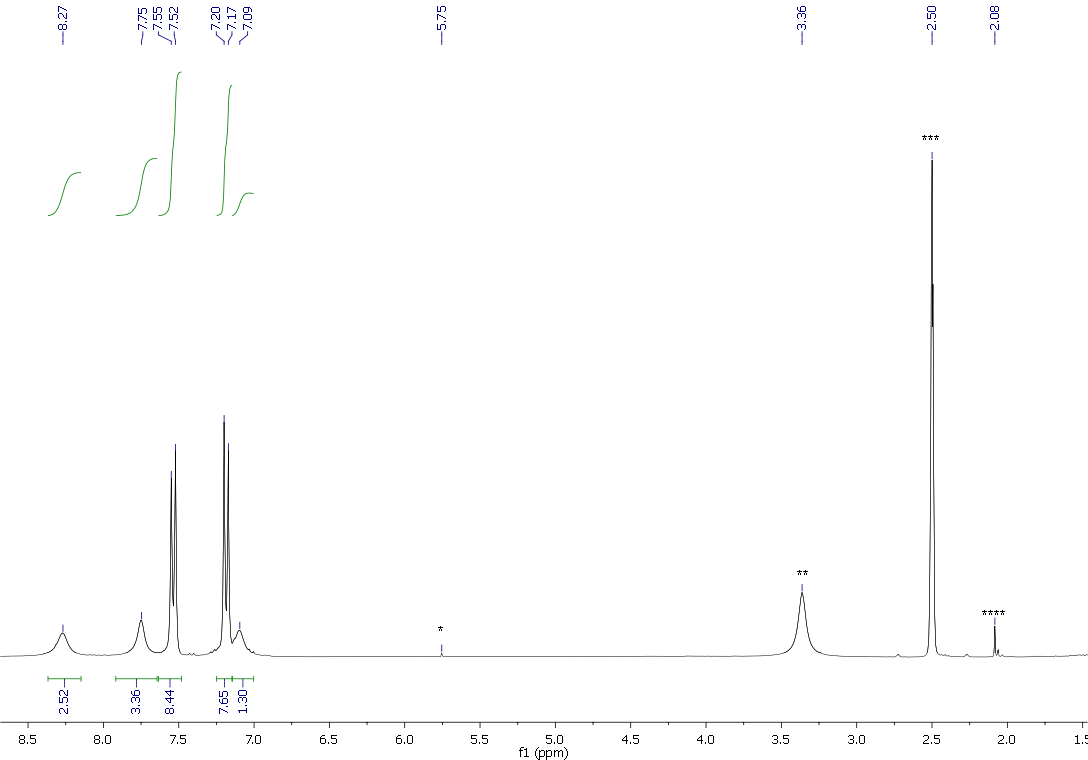


**Supplementary Figure 1.** ^1^H NMR spectrum of **TIPE** in DMSO-d_6_. Solvent residue: CH_2_Cl_2_(*), H_2_O(**), DSMO(***), Acetone(****).


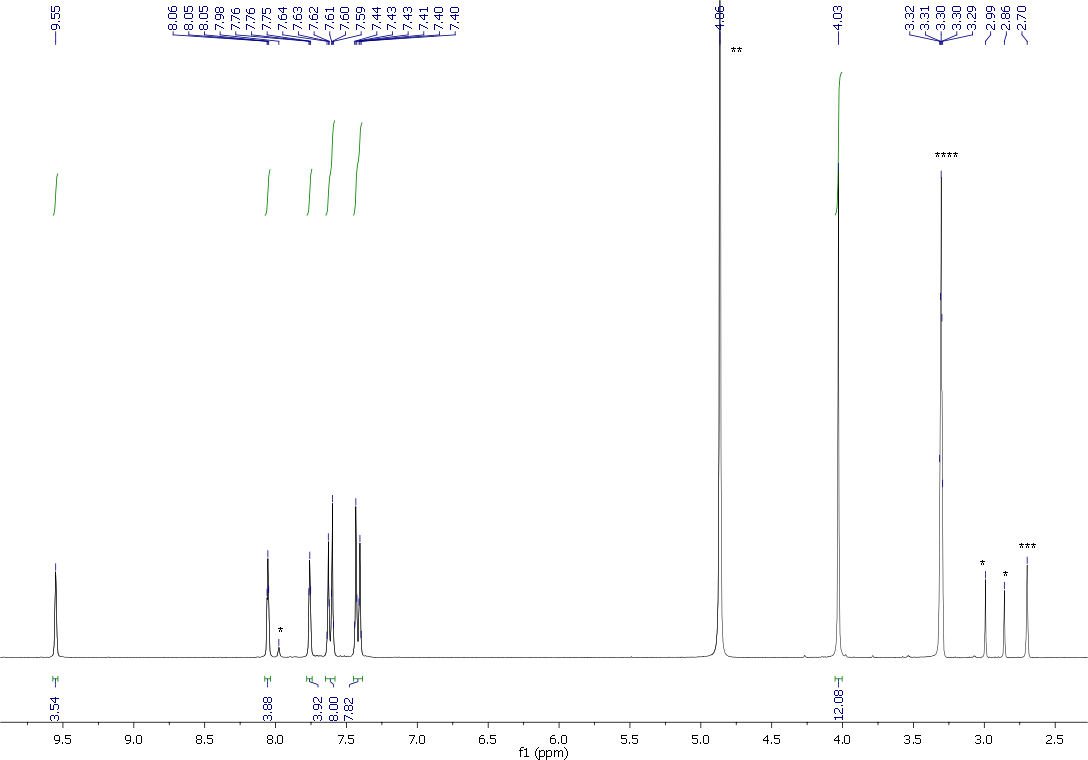


**Supplementary Figure 2.** ^1^H NMR spectrum of compound **TPE-Im** in CD_3_OD. Solvent residue: DMF(*), H_2_O(**), DSMO(***), CH_3_OH(****).

*
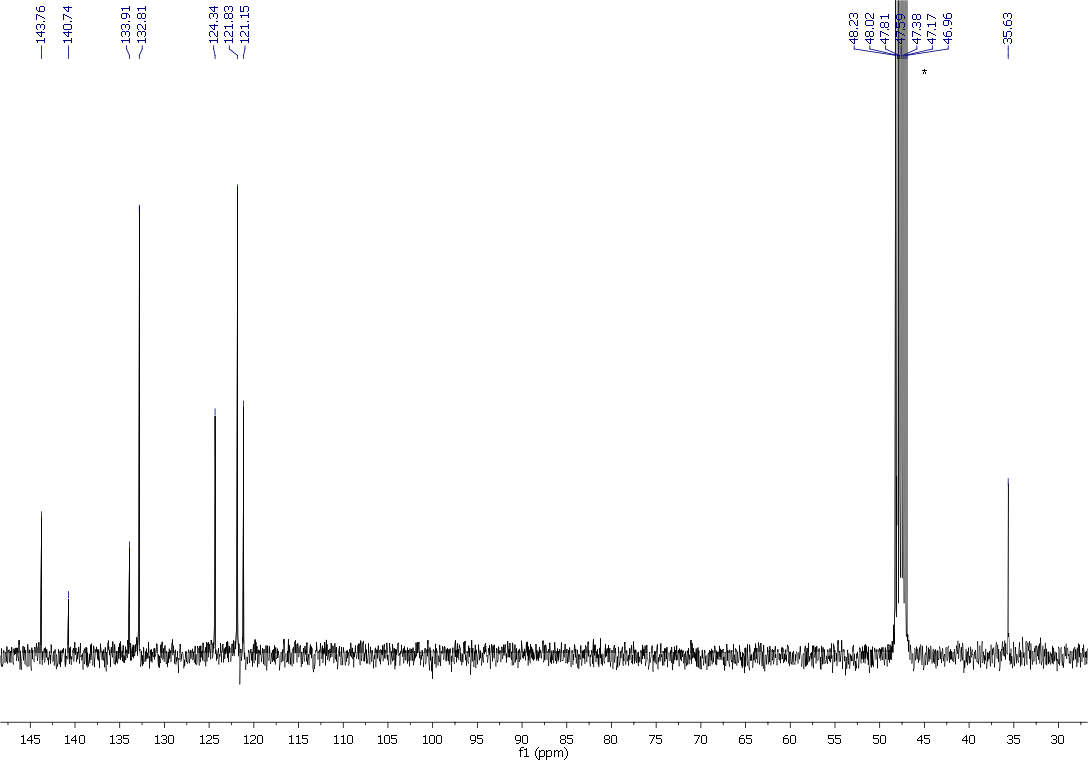
*

**Supplementary Figure 3.** ^13^C{^1^H} NMR spectrum of compound **TPE-Im** in CD_3_OD. Solvent residue: CH_3_OH(*).

**Computational details.** DFT calculations were performed on the tetracationic form of **TPE-Im** with the Gaussian 09 package (M. J. Frisch et al, 2016) using B3LYP hybrid-exchange correlation functional (Becke, 1993) and the 6/31G* basis set.


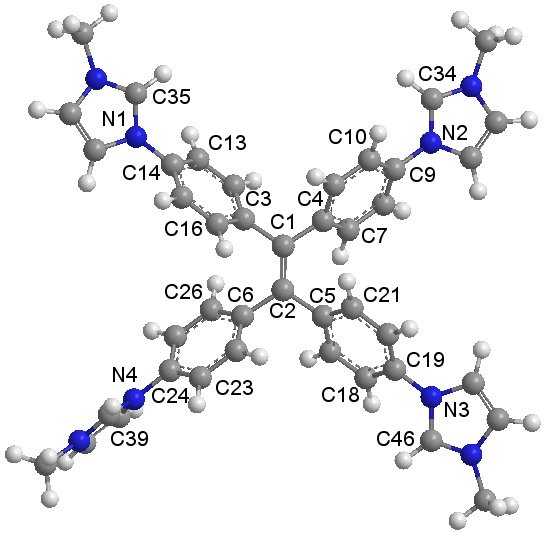


**Supplementary Figure 4.** DFT optimized structure of **TPE-Im.**

**Supplementary Table 1.** Selected torsion angle value found in **TPE-Im** optimized structure. Indications between brackets correspond to the torsion angle labelling in the article.

| Atom labels | Torsion angle (°) |
| --- | --- |
| C2-C1-C3-C16 [φ1] | 52.0 |
| C2-C1-C4-C7 [φ2] | 52.9 |
| C1-C2-C5-C21 [φ3] | 53.8 |
| C1-C2-C6-C26 [φ4] | 50.1 |
| C13-C14-N1-C35 | 46.3 |
| C10-C9-N2-C34 | 43.5 |
| C18-C19-N3-C46 | 43.2 |
| C23-C24-N4-C39 | 44.0 |


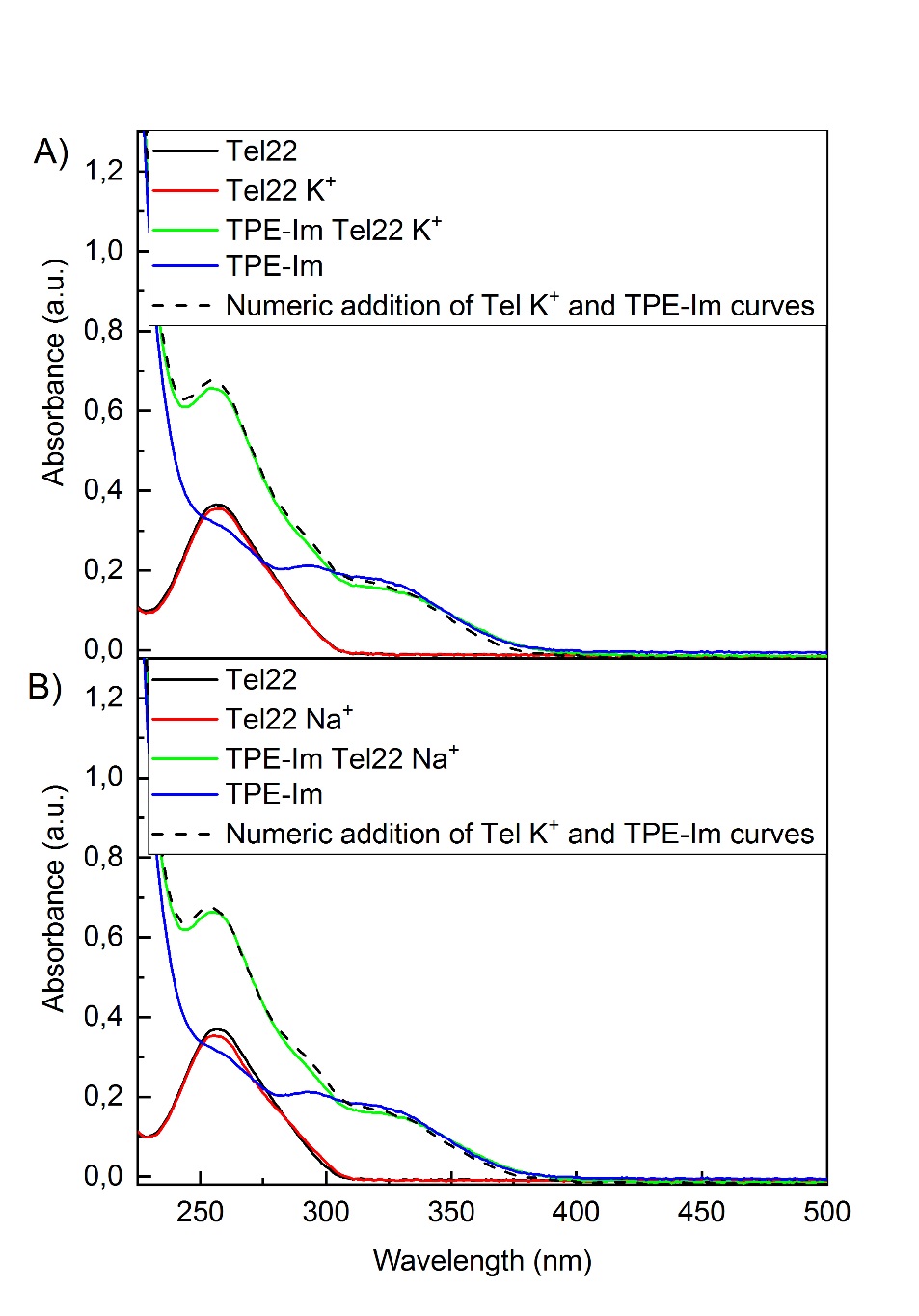


**Supplementary Figure 5.** UV-visible absorption spectra of A) **TPE-Im:Tel22** in TE buffer + 100mM KCl; B) **TPE-Im:Tel22** in TE buffer + 100mM NaCl. The molar ratio for A) and B) is 5:1 in **TPE-Im:Tel22.**


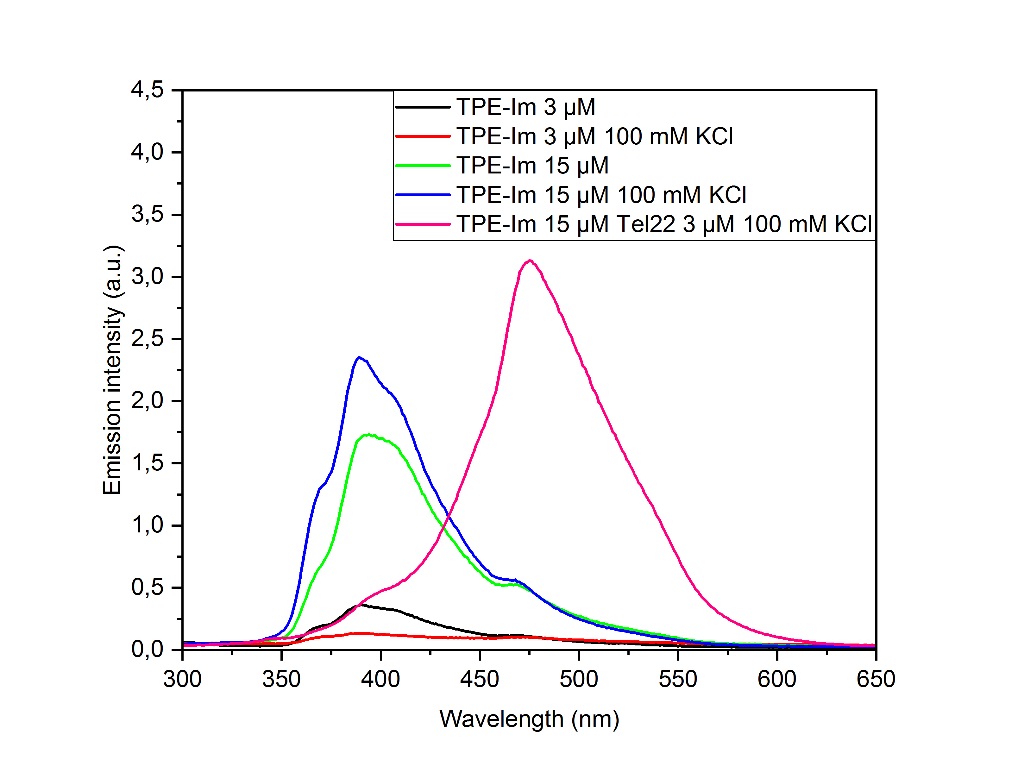


**Supplementary Figure 6.** Emission spectra of **TPE-Im** (λ_exc_ = 265 nm) at different concentrations with (black and green curve) or without KCl (red and blue curve) in comparison with the fluorescence of **TPE-Im** in the presence of **Tel22** and KCl at 5:1 ratio in **TPE-Im:Tel22** (pink curve).

**Supplementary Figure 7.** Emission spectra of **TPE-Im** (λ_exc_ = 265 nm) at 2 μM in a in physiological environment buffer.


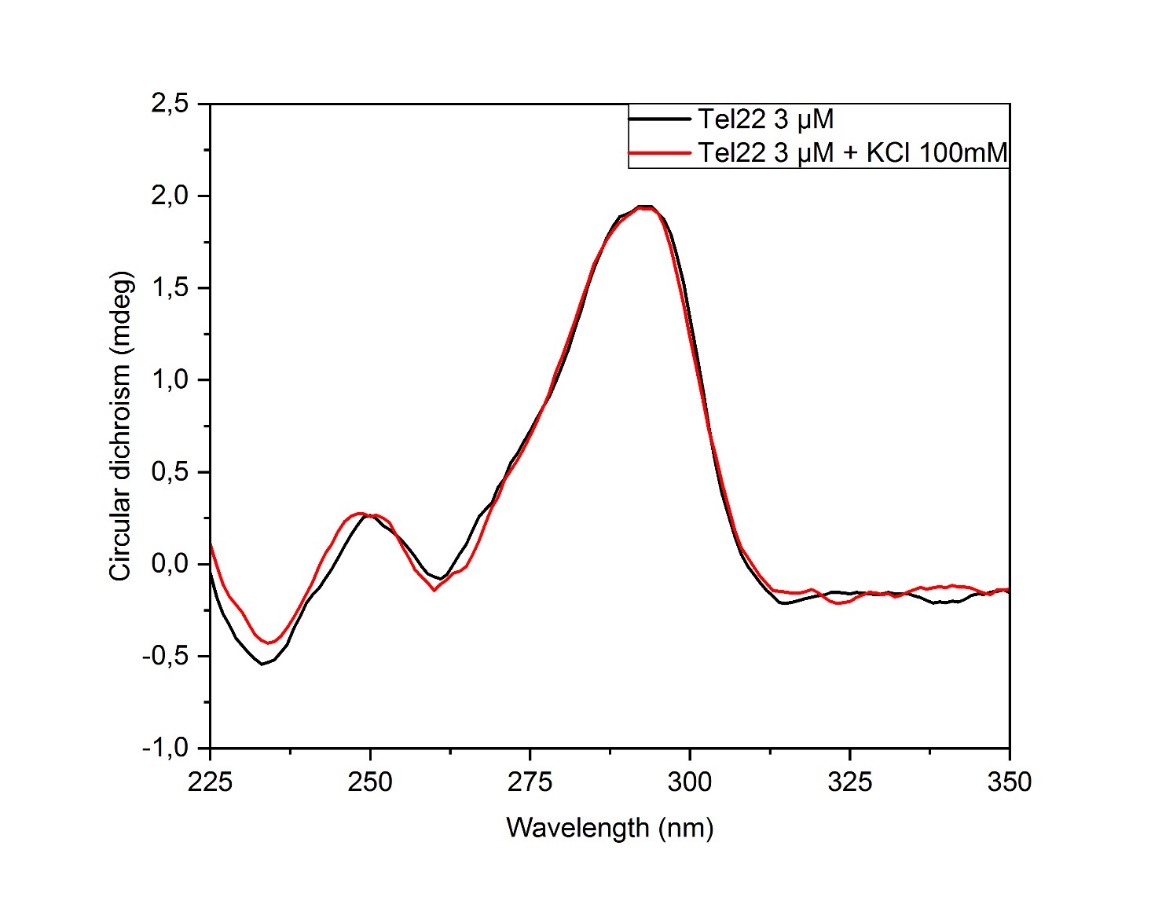


**Supplementary Figure 8.** CD Spectra of **Tel22** at 3µM in TE buffer.

**Supplementary Figure 9.** Emission spectra of **TPE-Im** (λ_exc_ = 265 nm) in the presence of human telomeric DNA sequence (**Tel22**) or double stranded DNA (**dsR_20_**) at different molar ratio (1:1 TPE-DNA in black and green; 5:1 TPE-DNA in red and blue).

**
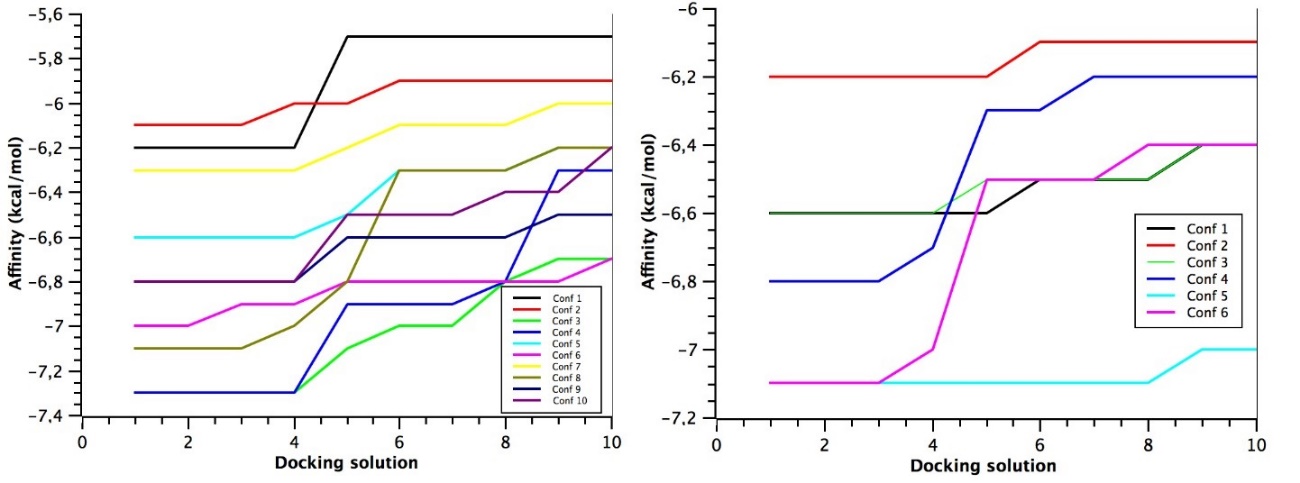
**

**Supplementary Figure 10.** Affinity scores of the 10 recorded docking solutions for each conformations of G4(2hy9) (left) and G4(143d) (right).

**Supplementary Table 2.** Statistical analysis of the recorded docking calculations

Statistical analysis of the 10 most stable docking solutions for each conformation of the 2HY9 G4 target.

| Target conformation  (2hy9) | Average affinity (kcal.mol^-1^) | Minimal affinity (kcal.mol^-1^) | Maximal affinity (kcal.mol^-1^) | RMSD  (Å) |
| --- | --- | --- | --- | --- |
| 1 | -5.9 | -5.7 | -6.2 | 12.18 |
| 2 | -6.0 | -5.9 | -6.1 | 6.91 |
| 3 | -7.1 | -6.7 | -7.3 | 1.85 |
| 4 | -6.9 | -6.3 | -7.3 | 5.32 |
| 5 | -6.4 | -6.2 | -6.6 | 8.61 |
| 6 | -6.9 | -6.7 | -7.0 | 5.77 |
| 7 | -6.2 | -6.0 | -6.3 | 16.88 |
| 8 | -6.6 | -6.2 | -7.1 | 7.72 |
| 9 | -6.7 | -6.5 | -6.8 | 8.95 |
| 10 | -6.6 | -6.2 | -6.8 | 2.13 |

Statistical analysis of the 10 most stable docking solutions for each conformation of the 143D G4 target.

| Target conformation  (143d) | Average affinity (kcal.mol^-1^) | Minimal affinity (kcal.mol^-1^) | Maximal affinity (kcal.mol^-1^) | RMSD  (Å) |
| --- | --- | --- | --- | --- |
| 1 | -6.5 | -6.4 | -6.6 | 2.68 |
| 2 | -6.2 | -6.1 | -6.2 | 8.10 |
| 3 | -6.5 | -6.4 | -6.6 | 8.90 |
| 4 | -6.5 | -6.2 | -6.8 | 5.32 |
| 5 | -7.1 | -7.0 | -7.1 | 3.07 |
| 6 | -6.7 | -6.4 | -7.1 | 3.65 |

**
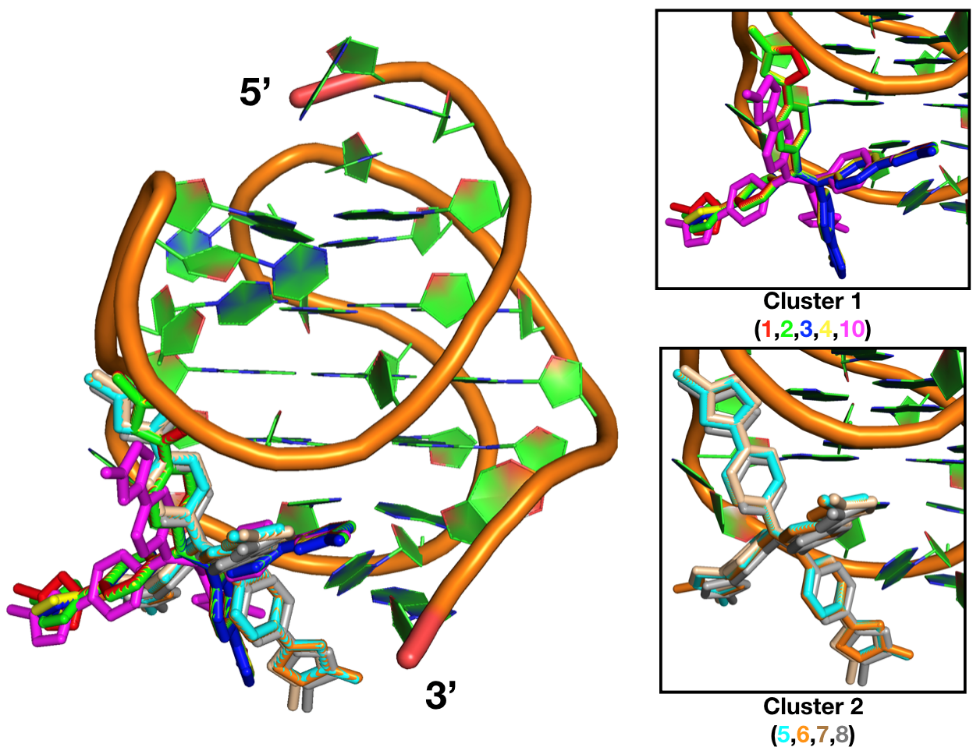
**

**Supplementary Figure 11.** Docking solutions of the **TPE-Im**/G4(2HY9, conformation 3)

**Supplementary Table 3.** Affinity score and RMSD of the 10 docking solutions between **TPE-Im** and G4(2hy9, conformation 3)

| Docking solution | Affinity (kcal.mol^-1^) | RMSD  (Å) |
| --- | --- | --- |
| 1 | -7.3 | 0.00 |
| 2 | -7.3 | 0.32 |
| 3 | -7.3 | 0.25 |
| 4 | -7.3 | 0.26 |
| 5 | -7.1 | 2.68 |
| 6 | -7.0 | 2.66 |
| 7 | -7.0 | 2.49 |
| 8 | -6.8 | 2.61 |
| 9 | -6.7 | 3.69 |
| 10 | -6.6 | 1.70 |


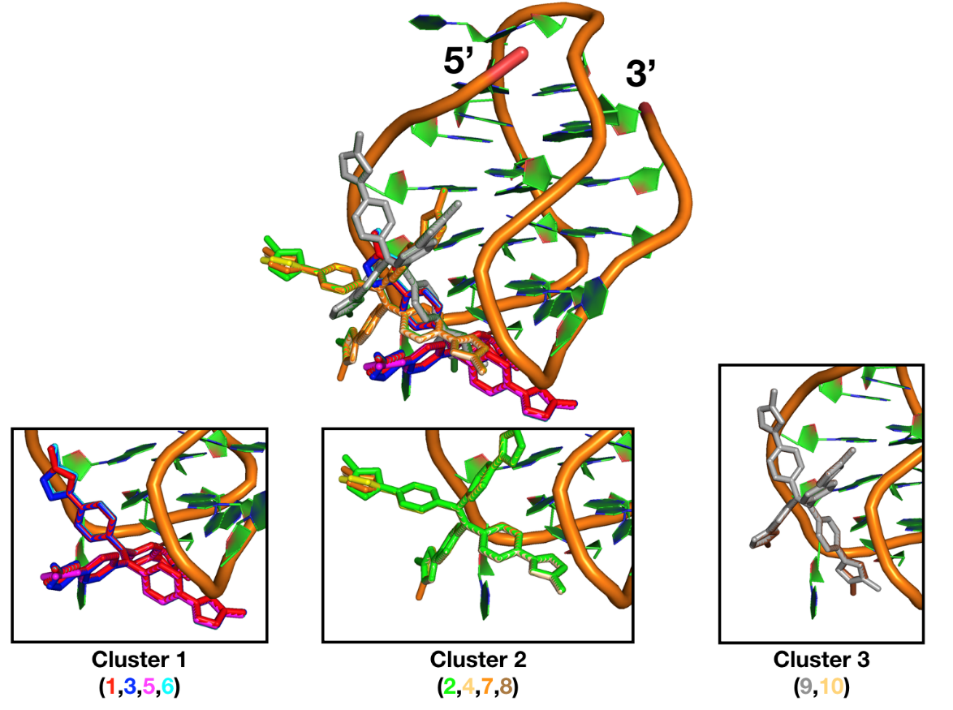


**Supplementary Figure 12.** Docking solutions of the **TPE-Im**/G4(143d, conformation 5)

**Supplementary Table 4.** Affinity score and RMSD of the 10 docking solutions between **TPE-Im** and G4(143d, conformation 5)

| Docking solution | Affinity (kcal.mol^-1^) | RMSD  (Å) |
| --- | --- | --- |
| 1 | -7.1 | 0.00 |
| 2 | -7.1 | 4.48 |
| 3 | -7.1 | 0.16 |
| 4 | -7.1 | 4.50 |
| 5 | -7.1 | 0.18 |
| 6 | -7.1 | 0.12 |
| 7 | -7.1 | 4.50 |
| 8 | -7.1 | 4.49 |
| 9 | -7.0 | 4.61 |
| 10 | -7.0 | 4.59 |

**Refererences**:

M. J. Frisch, G. W. Trucks, H. B. Schlegel, G. E. Scuseria, M. A. Robb, J. R. Cheeseman, G. Scalmani, V. Barone, G. A. Petersson, H. Nakatsuji, X. Li, M. Caricato, A. Marenich, J. Bloino, B. G. Janesko, R. Gomperts, B. Mennucci, H. P. Hratchian, J. V. Ortiz, A. F. Izmaylov, J. L. Sonnenberg, D. Williams-Young, F. Ding, F. Lipparini, F. Egidi, J. Goings, B. Peng, A. Petrone, T. Henderson, D. Ranasinghe, V. G. Zakrzewski, J. Gao, N. Rega, G. Zheng, W. Liang, M. Hada, M. Ehara, K. Toyota, R. Fukuda, J. Hasegawa, M. Ishida, T. Nakajima, Y. Honda, O. Kitao, H. Nakai, T. Vreven, K. Throssell, J. A. Montgomery, Jr., J. E. Peralta, F. Ogliaro, M. Bearpark, J. J. Heyd, E. Brothers, K. N. Kudin, V. N. Staroverov, T. Keith, R. Kobayashi, J. Normand, K. Raghavachari, A. Rendell, J. C. Burant, S. S. Iyengar, J. Tomasi, M. Cossi, J. M. Millam, M. Klene, C. Adamo, R. Cammi, J. W. Ochterski, R. L. Martin, K. Morokuma, O. Farkas, J. B. Foresman, and D. J. Fox, Gaussian, Inc., Wallingford CT, (2016). Gaussian 09, Revision A.02

Becke, A. D. (1993). Density‐functional thermochemistry. III. The role of exact exchange. *J. Chem. Phys.* 98, 5648–5652. doi:10.1063/1.464913.
